# Expression of risk genes linked to vitamin D receptor super-enhancer regions and their association with phenotype severity in multiple sclerosis

**DOI:** 10.3389/fneur.2022.1064008

**Published:** 2022-12-28

**Authors:** Sarah M. Orton, Amarpreet Sangha, Mehul Gupta, Kristina Martens, Luanne M. Metz, A. P. J. de Koning, Gerald Pfeffer

**Affiliations:** ^1^Faculty of Science and Technology, Mount Royal University, Calgary, AB, Canada; ^2^Department of Clinical Neurosciences, Cumming School of Medicine, Hotchkiss Brain Institute, University of Calgary, Calgary, AB, Canada; ^3^Department of Medical Genetics, Alberta Child Health Research Institute, Cumming of Medicine, University of Calgary, Calgary, AB, Canada

**Keywords:** multiple sclerosis, vitamin D deficiency, super-enhancers, vitamin D receptor (VDR), vitamin D

## Abstract

Multiple sclerosis (MS) is a chronic debilitating neurological condition with a wide range of phenotype variability. A complex interplay of genetic and environmental factors contributes to disease onset and progression in MS patients. Vitamin D deficiency is a known susceptibility factor for MS, however the underlying mechanism of vitamin D-gene interactions in MS etiology is still poorly understood. Vitamin D receptor super-enhancers (VSEs) are enriched in MS risk variants and may modulate these environment-gene interactions. mRNA expression in total of 64 patients with contrasting MS severity was quantified in select genes. First, RNA-seq was performed on a discovery cohort (10 mild, 10 severe MS phenotype) and ten genes regulated by VSEs that have been linked to MS risk were analyzed. Four candidates showed a significant positive association (GRINA, PLEC, PARP10, and LRG1) in the discovery cohort and were then quantified using digital droplet PCR (ddPCR) in a validation cohort (33 mild, 11 severe MS phenotype). A significant differential expression persisted in the validation cohort for three of the VSE-MS genes: GRINA (*p* = 0.0138), LRG1 (*p* = 0.0157), and PLEC (*p* = 0.0391). In summary, genes regulated by VSE regions that contain known MS risk variants were shown to have differential expression based on disease severity (p<0.05). The findings implicate a role for vitamin D super-enhancers in modulating disease activity. In addition, expression levels may have some utility as prognostic biomarkers in the future.

## Introduction

Multiple sclerosis (MS) is a chronic and debilitating autoimmune condition of the central nervous system (1). A complex interplay of genetic and environmental factors contributes to disease onset and progression in MS patients (2). Vitamin D deficiency is a known susceptibility factor for MS, however the underlying mechanism of vitamin D gene-environment interactions in MS etiology is still poorly understood (3).

There are several lines of evidence supporting the role of vitamin D in MS susceptibility, including higher MS prevalence in regions with lower annual UVB exposure (4–7), and migrant studies showing changes to MS risk based on latitude changes (8–10). Increased actinic sun damage, and reporting more time outdoors during childhood/adolescence, have both been associated with reduced risk of MS (11–13). Large prospective studies measuring vitamin D status prior to MS onset showed that participants with low serum vitamin D levels were at greater risk of developing MS (14–16). Clinical studies have reported that vitamin D supplementation in MS patients lowered disease relapse risk (17–21), while lower vitamin D status at MS onset is associated with worse cognitive function and neuronal integrity (22). MS genetic risk loci, identified by GWAS, are enriched with binding sites for the vitamin D receptor (VDR) (23), and several MS risk variants have been localized to VDR binding sites (24).

Recent *in silico* evidence suggests that vitamin D receptor (VDR) super-enhancers (VSE) may be influenced by nearby MS risk SNPs, thereby impacting the activity of these VSEs in relation to disease (25). Vitamin D exerts its biological effects when the active hormone [1,25-dihydroxyvitamin D, 1,25(OH)_2_D] binds VDR, which then acts as a transcription factor upon binding to VDR response elements in target genes (26). These VDR target genes are often associated with clusters of VDR binding sites that form super-enhancers, regulatory genomic regions consisting of dense clusters of enhancers (27). VSEs contain clusters of VDR binding sites, and loop to hundreds of promoter regions, upregulating vitamin D target genes (27).

Lu et al. (25) identified specific genes near VSE regions that overlap with MS risk variants. We investigated the expression of ten VSE-MS-linked genes in a cohort of 64 persons with MS representing the two extremes of disease activity, to assess whether expression differences were associated with mild vs. severe MS phenotype.

## Methods

### Ethics statement

The Conjoint Health Research Ethics Board (REB17-1193) at the University of Calgary provided approval for this study. Written informed consent was obtained from all participants.

### Study population and design

Selection criteria and experimental methodology have been described previously (28). Briefly, study participants (*n* = 64) representing a range of phenotypic severity were selected from a large prospective cohort consisting of 2,831 participants recruited for an ongoing prospective cohort study at the University of Calgary MS Clinic in Calgary, Alberta. Patients were identified according to their age-related MS severity (ARMSS) scores over three or more clinical visits. 600 patients with the lowest ARMSS scores, and 600 patients with the highest ARMSS scores were designated as mild and severe phenotypes, respectively. From this subset, 64 patients were recruited for this pilot project: 43 patients with a mild phenotype (mean ARMSS score 1.34, range 0.06–3.08) and 21 patients with a severe phenotype (mean ARMSS score 6.90, range: 5.73–9.94). Clinical parameters were assessed to ensure relative homogeneity between the two groups, including age of MS onset, age at recruitment, sex, and use of disease modifying therapies. Mann-Whitney-Wilcoxon test for ordinal variables and Chi-square test for nominal variables were used to assess statistical significance.

A two-phase discovery and validation approach was employed. Candidate MS-VSE genes were identified in a discovery cohort consisting of 20 patients (10 mild, 10 severe phenotype) in which transcriptome sequencing (RNA-seq) was conducted. Genes demonstrating significant differential expression between mild and severe phenotypic subgroups were then retained and quantified in a validation cohort using digital droplet PCR (ddPCR).

### RNA isolation and quality control

The Paxgene RNA Purification Kit was utilized to isolate total RNA from whole blood samples of recruited participants. Sample integrity was verified by fluorimetry using an RNA-specific dye (Qubit fluorimeter), and RIN analysis with an Agilent TapeStation 2200 (RIN score range 6.4–8.4).

### High throughput RNA-sequencing

Low Sample protocol was followed using the #20020596 TruSeq Stranded Total RNA Library Preparation Kit (H/M/R) on 500 ng of each RNA sample. RiboZero magnetic beads were used to remove rRNA and keep the Illumina TruSeq i7 indices in the RNA samples. The fragments present in the RNA samples were enriched *via* 15 cycles of PCR amplification. The resulting libraries were validated by TapeStation analysis and qPCR using the Kapa qPCR Library Quant Kit for Illumina. Following validation, the libraries were then pooled and sequenced on an Illumina NextSeq 500 sequencer. The pool of 20 samples underwent single-end sequencing, which involved three consecutive high-output NextSeq V2 sequencing runs, consisting of 75 cycles for each run. Paired-end sequencing was also conducted on the 20-sample pool using a single 150 cycle (2 × 75 bp) high-output NextSeq V2 sequencing run. The single-end sequencing and paired-sequencing yielded an average of 80 million clusters PF per sample and 26 million clusters PF per samples, respectively.

### Bioinformatics analysis

Transcriptomic data was processed by aligning counts to the EnsEMBL GRCh38.p12 genome reference assembly utilizing FeatureCounts. Differential expression between mild and severe phenotype patients was then conducted using the DeSeq2 R Package. Ten candidate genes were selected based on previous findings. Firstly, five genes regulated by VSEs which contain known MS risk variants were previously identified (25) and thus selected for inclusion: UBASH3B, IRF8, PLEC, PARP10, GRINA. The second set of genes overlap with MS risk variants and were under significant regulation by 1,25(OH)D *via* VSEs, with high expression levels (25): DENND6B, USP2, ASAP2, SEMA6B, LRG1. The targets that showed a statistically significant difference (Log2 fold change, *p* < 0.05, Wald test) were then retained for analysis in the validation cohort.

### Digital droplet PCR (ddPCR) validation analysis

Validation analysis were performed using digital droplet PCR due to its increased sensitivity and absolute quantification method compared to conventional PCR techniques. ddPCR custom primers were designed using the Ensembl database and Primer3 software, which generated sequences for forward and reverse primers (Supplementary Table 1). The Bio-Rad iScript cDNA Synthesis Kit and random hexamers were used to perform reverse transcription on the remaining 44 RNA samples. Based on the Bio-Rad QX200 ddPCR system protocol, custom primers designed for each candidate and Bio-Rad ddPCR EvaGreen Supermix was used. The expression of each candidate was normalized to the expression of *HPRT1* and *B2M* given their relatively consistent expression profiles. The normalized counts for each candidate were then analyzed using a one-tailed *t*-test in GraphPad Prism 8.4.1 to determine if a significant difference in expression existed between the mild and severe MS phenotypes.

## Results

Clinical characteristics of the study participants are listed in Table 1. There were large differences in ARMSS scores between the mild and severe phenotype groups, as expected due to study design (Table 1). Across all study participants, the mean ARMSS scores were 1.34 and 6.90, for the mild and severe phenotypes, respectively. Other variables, including the age at onset, age at recruitment, and sex (% female) were not significantly different between mild and severe phenotypes (Table 1). The severe phenotype groups in both cohorts consisted of only female patients (Table 1). The number of participants in each group receiving at least one of the MS disease modifying therapies, as well as the types of treatments, did not differ significantly between the groups; 58.1% in the mild group, 61.9% in the severe group (*data not shown*).

**Table 1:** Clinical characteristics of included study participants.

	**Discovery cohort**		
	**Mild phenotype** **(*n* = 10)**	**Severe phenotype** **(*n* = 10)**	***p*** **-value**
Mean age (range)	53.4 (32.4–67.4)	52.1 (39.6–59.5)	0.7133
Mean age at MS Onset (range)	35.6 (20.9–46.2)	28.7 (14.8–46.1)	0.7649
Sex (% female)	70%	100%	0.2105
Mean ARMSS score (range)	1.177 (0.060–2.194)	7.255 (6.187–9.401)	< 0.0001
	**Validation cohort**		
	**Mild phenotype** **(*****n*** = **33)**	**Severe phenotype** **(*****n*** = **11)**	***p*** **-value**
Mean age (range)	57.8 (45.3–70.1)	54.6 (34.4–67.1)	0.4480
Mean age at MS Onset (range)	33.9 (16.6–56.2)	32.2 (14.6-47.3)	0.09692
Sex (% female)	60.6%	100%	0.0189
Mean ARMSS score (range)	1.864 (0.225–3.017)	6.518 (5.804–7.891)	<0.0001

**Figure 1:**
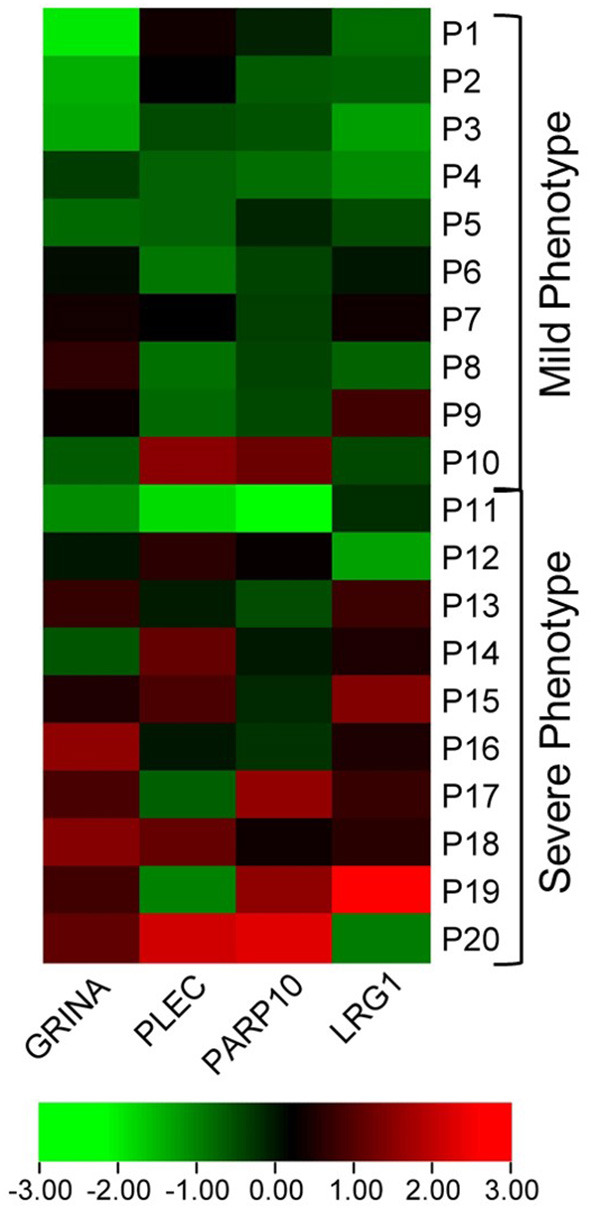
Heat map visualizing differential expression in MS-VSE genes in the extremes of MS phenotype discovery cohort. The color legend illustrates the coding by log-fold difference in expression (P1 = Patient 1).

**Figure 2:**
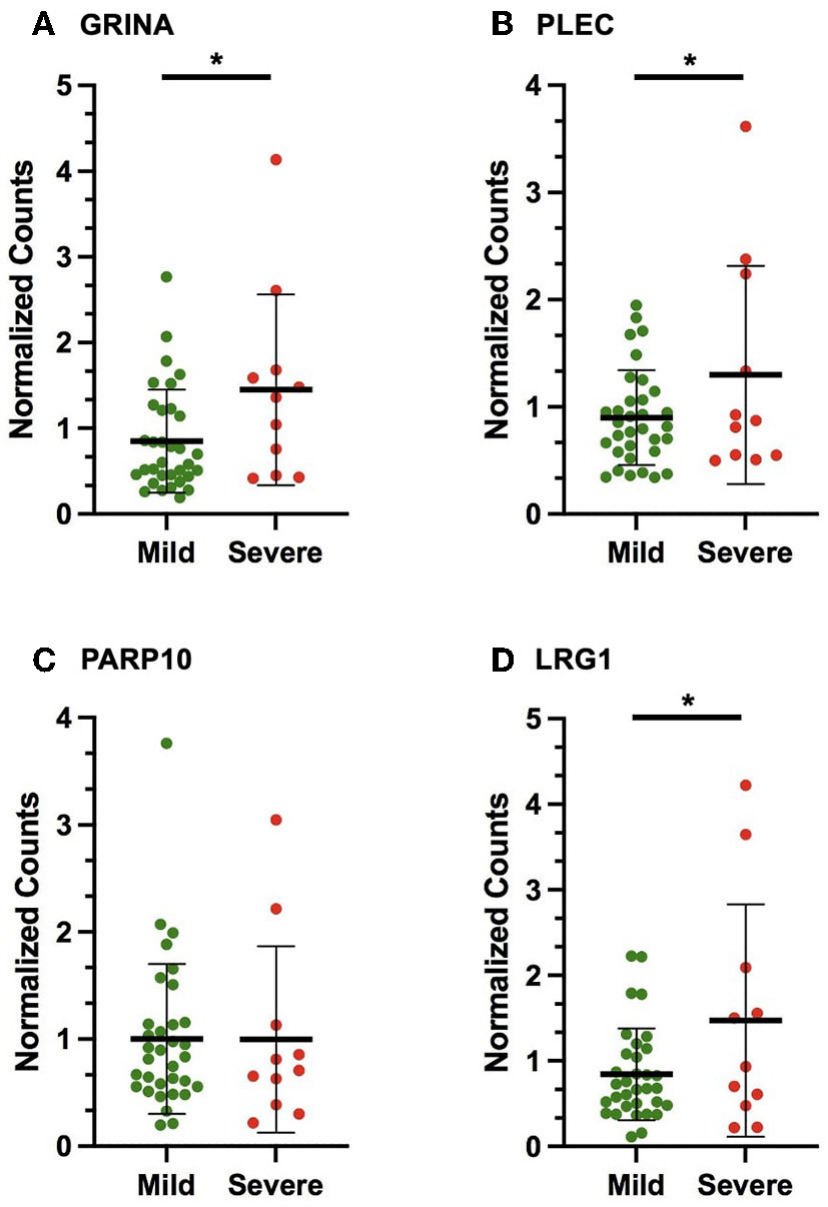
Scatter plots displaying the number of gene copies normalized to the expression of HPRT1 and B2M (normalized counts) in the validation cohort for the following VSE-MS-linked candidates: **(A)** GRINA, **(B)** PLEC, **(C)** PARP10, **(D)** LRG1. The mean and standard deviation are shown. The asterisks (*) indicate statistically significant results (*p* < 0.05).

In the discovery cohort, whole transcriptome sequencing was conducted for the ten candidate MS-linked-VSE genes selected based on prior evidence (Figure 1). Mean expression levels were analyzed for significant differences in the mild vs. severe phenotype groups (≥ 2-fold-change, *p* < 0.05). Four of the candidate MS-linked-VSE genes GRINA, PLEC, PARP10, and LRG1 were significantly upregulated in the severe phenotype MS subgroup (Table 2), and thus were evaluated in the validation cohort (*n* = 44) using custom-designed ddPCR assays. This analysis demonstrated three genes that were differentially expressed between MS phenotypes including GRINA, PLEC, and LRG1 (Figure 2).

**Table 2:** Differential gene expression between mild and severe MS phenotypes in the discovery and validation cohorts; mean counts were determined by RNA-seq (number of sequenced fragments) and by ddPCR (number of gene copies, normalized to HPRT1 and B2M expression), respectively.

	**Discovery cohort**				**Validation cohort**			
**MS-VSE candidates**	Mean counts [Standard deviation]				Mean counts [standard deviation]			
	Mild	Severe	log2FC	*p*-value, differential transcript levels	Mild	Severe	log2FC	*p*-value, normalized to HPRT1 and B2M
**GRINA**	832 [268]	1,240 [368]	0.57	0.0034^*^	0.85 [0.60]	1.45 [1.11]	0.77	0.0138^*^
**PLEC**	2,885 [1,019]	3,608 [1,606]	0.32	0.0006^*^	0.90 [0.44]	1.29 [1.01]	0.52	0.0381^*^
**PARP10**	717 [255]	1,071 [652]	0.58	0.0028^*^	1.00 [0.70]	0.99 [0.86]	−0.01	0.4925
**LRG1**	529 [140]	780 [348]	0.78	0.0063^*^	0.84 [0.54]	1.47 [1.36]	0.81	0.0157^*^

The asterisks (^*^) indicate statistically significant results (*p* < 0.05).

## Discussion

This pilot study compared expression of MS-VSE genes in mild vs. severe MS phenotypes, in order to elucidate a potential role for vitamin D in MS etiology, as well as identifying potential future biomarkers for differentiating these phenotype subgroups. Vitamin D super-enhancers in MS is a relatively new area of research and this is the first study, to our knowledge, to directly investigate the putative role of VSE-linked genes associated with MS risk SNPs in MS phenotypic severity.

Vitamin D deficiency has long been implicated in MS susceptibility, yet the exact vitamin D gene-environment or epigenetic interactions in MS etiology remain unclear. A better understanding may at best help in prevention or modulation of disease severity, or at least create awareness of the importance of vitamin D sufficiency. Active vitamin D, 1,25(OH)D exerts its biological effects by binding to the VDR and altering gene expression. Vitamin D super-enhancers contain multiple VDR binding sites, which then loop to transcription start site of target gene(s) to stimulate transcription (29). VSEs are believed to have more pronounced effects on gene expression than traditional enhancers (and many are signal-inducible *via* 1,25(OH)D exposure (26, 27). Recent in silico evidence identified specific genes with VSE regions overlapping with MS risk variants as well as VSE genes overlapping with MS risk variants (25). We compared the expression of ten of these identified candidates in our cohort. It is hypothesized that MS risk SNPs within VSEs, or genes associated with VSEs may alter the VSE activity to impact disease risk and activity (30).

RNA-sequencing and ddPCR were used to assess differential expression of select candidates. Three VSE genes (PLEC, GRINA, LRG1) that were previously identified as linked to MS variants (25) showed consistent differences in expression between the mild vs. severe phenotype groups in both the discovery and confirmation cohorts. It should be noted that for neurodegenerative diseases, even small fold-changes in expression levels can relate to disease development and activity (24, 31, 32). Interestingly, a significant increase in transcription levels was associated with these VSE-genes in the severe phenotype. The findings implicate a role for vitamin D super-enhancers in modulating disease *via* environment-gene interactions. In addition, expression levels may have some utility as prognostic biomarkers in the future.

The molecular mechanisms involved in VSE regulation of gene expression and its impact on MS has not been studied. GRINA encodes a glutamate ionotropic receptor N-methyl-D-aspartate (NMDA)-type subunit associated protein 1, expressed on the postsynaptic membrane of neuronal synapses (33). The receptor is an excitatory glutamate-gated ion channel found throughout the body (34). Excessive glutamate at the sites of demyelination and overstimulation of glutamate receptors leading to neuronal death have been reported (35, 36). The PLEC gene encodes the cytoskeleton plectin protein, which is found in many tissues, including nervous tissue (37, 38). The degradation of cerebral tissue integrity and elasticity in MS patients, especially in glial cells (39), may be affected. LRG1 encodes the leucine-rich alpha-2-glycoprotein, which has recently been reported to promote angiogenesis in the brain (40, 41).

While it is hypothesized that VSE-related MS risk variants may lead to changes in the epigenomic landscape involved in MS pathogenesis, we cannot rule out that the converse is true—it may be that disease severity affects VDR related changes in chromatin accessibility and thus regulation of gene expression. It may also be a combination of both effects. Studying expression in individuals with first de-myelinating events, and/or longitudinally, would be needed to better support any potential cause-and-effect relationship.

Given the small sample size in this pilot study, follow up in larger phenotype cohorts or case-control designs, will be useful to validate a role for VSE genes in MS risk and severity, as well as to explore a potential role as clinically useful biomarkers. In particular, a comparison of mild cases vs. healthy controls and severe cases vs. healthy controls would be warranted in this validation. Furthermore, the severe phenotype group in the validation cohort contained exclusively females, and obtaining larger sample sizes with more males are warranted to confirm the findings. While the cohorts do not differ in the baseline demographic characteristics, we acknowledge the limitation on the results of not having more precise 1:1 propensity score matching between the individuals in the discovery and validation cohorts.

Another potential limitation of this study involves the use of whole blood, which has both advantages and disadvantages, as we have discussed previously (28). Many recent publications studying MS biomarkers have used whole blood (42). Other blood sample types (serum, plasma) require more onerous standardization to avoid variability in sample contents introduced (43). We believe that whole blood remains a reasonable choice for gene expression studies in MS because the collection is easily standardized, and there is precedent from numerous prior studies. Recent publications continue to demonstrate the important role of peripheral blood cells in the pathogenesis of MS (44, 45). Nonetheless, future studies extracting transcripts from different cell types could help determine if the results are more generalizable, given that the genomic binding pattern of VDR can be variable between tissues (29). While our findings support a link between VSE genes and MS risk variants, the mechanisms involved have not been studied. Extension to murine EAE models could explore functional effects.

The study of vitamin D super-enhancers, and their effect on human health, is a relatively new and growing area of study. There are very limited publications in this area for the field of multiple sclerosis. The candidate genes investigated may be important players in the interaction between the environment (vitamin D) and genetic risk associated with multiple sclerosis.

## Data availability statement

The original contributions presented in the study are included in the article/Supplementary material, further inquiries can be directed to the corresponding author/s.

## Ethics statement

The studies involving human participants were reviewed and approved by University of Calgary Conjoint Health Research Ethics Board (REB17-1193). The patients/participants provided their written informed consent to participate in this study.

## Author contributions

SO: conceived study and design, lab work, data analysis, data interpretation, manuscript writing, and manuscript editing. AS: lab work, data collection, data analysis, and manuscript writing. MG: data collection, data analysis, and manuscript editing. KM: technical assistance and experiments. LM: cohort collection and study design. AK: cohort collection, study design, and data analysis. GP: cohort collection, study design, data interpretation, and manuscript revision. All authors reviewed and approved the final manuscript version.
