# Supplementary material for: Expression of risk genes linked to vitamin D receptor super-enhancer regions and their association with phenotype severity in multiple sclerosis
Source: Front Neurol. 2022 Dec 28;13:1064008. doi: 10.3389/fneur.2022.1064008 (PMC9832371; doi:10.3389/fneur.2022.1064008)
Supplement: Supplementary file 1 [file Table_1.pdf]

Supplementary Table S1. Sequences for custom primers used for ddPCR in the validation cohort.

Gene	Primer Sequences		%GC Content	Exon number
**LRG1**	Forward	CAGACAGCGACCAAAAAGC	52.6	1-2
	Reverse	GGAGAGGTGCAATTCTTGGA	60.2	2
**PLEC**	Forward	GTACGCCAAGCTGCTGAACT	55.0	15-16
	Reverse	CAGCATTTTGGAGCTCCTTG	50.0	17
**PARP10**	Forward	TATGACTGGCTTTCGGCTCT	50.0	4-5
	Reverse	CTTCAAGGCCTGTGTCCAAC	55.0	5
**GRINA**	Forward	CCAGGACAAGACCCTGACTC	60.0	1-2
	Reverse	GGACAAAGCCCTTCACCTC	57.9	3
